# Acute sympathetic activation blunts the hyperemic and vasodilatory response to passive leg movement in young healthy males

**DOI:** 10.14814/phy2.70180

**Published:** 2025-02-27

**Authors:** Brady E. Hanson, Joshua F. Lee, Ryan S. Garten, Zachary Barrett‐O'Keefe, Gwenael Layec, Bradley A. Ruple, D. Walter Wray, Russell S. Richardson, Joel D. Trinity

**Affiliations:** ^1^ Geriatric Research, Education, and Clinical Center George E. Whalen VA Medical Center Salt Lake City Utah USA; ^2^ Department of Internal Medicine, Division of Geriatrics University of Utah Salt Lake City Utah USA; ^3^ Department of Kinesiology and Health Sciences Virginia Commonwealth University Richmond Virginia USA; ^4^ Department of Cardiovascular Medicine Mayo Clinic Rochester Minnesota USA; ^5^ Mayo Clinic School of Health Sciences, Mayo Clinic College of Medicine and Science Mayo Clinic Rochester Minnesota USA; ^6^ School of Health and Kinesiology University of Nebraska Omaha Omaha Nebraska USA; ^7^ Department of Nutrition and Integrative Physiology University of Utah Salt Lake City Utah USA

**Keywords:** autonomic physiology, blood flow regulation, vascular function

## Abstract

Heightened muscle sympathetic nerve activity (MSNA) contributes to impaired vasodilatory capacity and vascular dysfunction associated with aging and cardiovascular disease. The contribution of elevated MSNA to the vasodilatory response during passive leg movement (PLM) is not fully understood. This study tested the hypothesis that elevated MSNA diminishes the vasodilatory response to PLM in healthy young males (*n* = 11, 25 ± 2 yr). Post exercise circulatory occlusion (PECO) following 2 min of isometric handgrip (HG) exercise performed at 25% (ExPECO 25%) and 40% (ExPECO 40%) maximum voluntary contraction was used to incrementally engage the metaboreceptors and augment MSNA. Control trials were performed without PECO (ExCON 25% and ExCON 40%) to account for changes due to HG exercise. PLM was performed 2 min after exercise and hemodynamics were assessed. MSNA was recorded by microneurography in the peroneal nerve (*n* = 8). Measures of MSNA (i.e., burst incidences) increased during ExPECO 25% (+15 ± 5 burst/100 bpm) and ExPECO 40% (+22 ± 4 burst/100 bpm) and returned to pre‐HG levels during ExCON trials. Leg vascular conductance (vasodilation) during PLM was reduced by 16% and 44% during ExPECO 25% and ExPECO 40%, respectively. These findings indicate elevated MSNA attenuates the vasodilatory response to PLM and the magnitude of reduction in vasodilation during PLM is graded with the degree of sympathoexcitation.

## 1 INTRODUCTION

The sympathetic nervous system plays a critical role in the short‐ and long‐term regulation of blood pressure and vascular conductance by directly altering vasomotor tone (Charkoudian & Rabbitts, 2009; Fairfax et al., 2013; Wallin & Charkoudian, 2007). Derangements in sympathetic nervous system activity, characterized by heightened muscle sympathetic nerve activity (MSNA), negatively affect vasomotor tone and inhibit vasodilatory capacity. Several cardiovascular diseases including hypertension (Grassi, 1998; Yamada et al., 1989) and heart failure (Leimbach Jr. et al., 1986; Notarius et al., 1999), as well as healthy aging (Dinenno et al., 2000; Fagius & Wallin, 1993), are associated with augmented MSNA and concomitant vascular dysfunction. Moreover, both MSNA and vascular function provide important and predictive insight into future adverse cardiovascular events (Green et al., 2011; Neunteufl et al., 2000). The link between vascular dysfunction and elevated MSNA appears to be more than coincidental, as acute elevations in MSNA result in impaired macrovascular function as measured by brachial artery flow mediated dilation (FMD) (Dyson et al., 2006; Hijmering et al., 2002; Lind et al., 2002). Previously, our group superimposed arm exercise on passive leg movement (PLM) and reported reduced PLM‐induced vasodilation suggesting that heightened exercise‐induced MSNA restricts vasodilation during PLM (Venturelli et al., 2022). Clearly, the axis of sympathetic nervous system activity and vascular function is important in the integration and regulation of cardiovascular health. It should be noted, however, that although elevated MSNA may indeed contribute to the observed alteration in PLM, exercise induces other modulatory responses including increases in central command as well as mechanoreceptor and metaboreceptor activation that directly impact hemodynamic regulation (Wan et al., 2023). Thus, how elevated MSNA influences the vasodilatory response to PLM independent of such systemic cardiovascular responses during exercise is not fully understood.

We have previously used PLM to noninvasively assess vascular function (Trinity et al., 2012). PLM provides a reductionist approach to assess movement‐induced hyperemia and vasodilation without the confounding increase in oxygen demand and metabolic vasodilation (McDaniel, Fjeldstad, et al., 2010; Trinity et al., 2011). Nearly 80% of the hyperemic and vasodilatory response to PLM is nitric oxide (NO) dependent signifying the utility of this method to provide a valid assessment of NO‐mediated vascular endothelial function (Mortensen et al., 2012; Trinity et al., 2012, 2021). Moreover, the hyperemic and vasodilatory responses to PLM are significantly reduced by aging, heart failure, and spinal cord injury indicating that PLM provides a sensitive assessment of vascular function across a range of conditions and groups (Groot et al., 2022; Hayman et al., 2010; Lew et al., 2021; McDaniel, Hayman, et al., 2010). As previously described, acute and chronic elevations in MSNA are associated with reductions in vascular function (DeLorey, 2021; Hijmering et al., 2002; Venturelli et al., 2022). Using a pharmacological approach, we recently reported that increasing α‐adrenergic activation attenuates the PLM response (Fermoyle et al., 2023). Pharmacological approaches provide valuable mechanistic insight but may override the natural physiological state associated with elevated MSNA.

Therefore, this study sought to determine how acute alterations in MSNA impact the vasodilatory and hyperemic responses to PLM. To this end, post exercise circulatory occlusion (PECO) was used following static isometric handgrip exercise at two exercise intensities to isolate the metaboreflex and incrementally augment MSNA during PLM (Boushel, 2010; Fisher et al., 2015). We directly tested the hypothesis that within a cohort of young, generally healthy males, graded increases in MSNA would progressively reduce the vasodilatory response to PLM revealing a critical role of sympathetic control in the PLM‐induced vasodilatory response.

## 2 METHODS

### 2.1 Subjects

Eleven healthy males (25 ± 2 y, 178 ± 2 cm, 72 ± 3 kg) volunteers were enrolled in this research study. Subjects were nonsmokers, not taking any prescription medications, and were free from overt cardiovascular disease. For all study visits, subjects were asked to refrain from consuming alcohol‐ and caffeine‐containing beverages and strenuous exercise for 24 h prior to each visit. Protocol approval and written informed consent were obtained according to the University of Utah and Salt Lake City Veteran's Administration Medical Center (VAMC) Institutional Review Boards, in accordance with the principles outlined in the *Declaration of Helsinki*. All data collection took place at the Salt Lake City VAMC Geriatric Research, Education, and Clinical Center in the Utah Vascular Research Laboratory.

### 2.2 Experimental protocol

**FIGURE 1:**
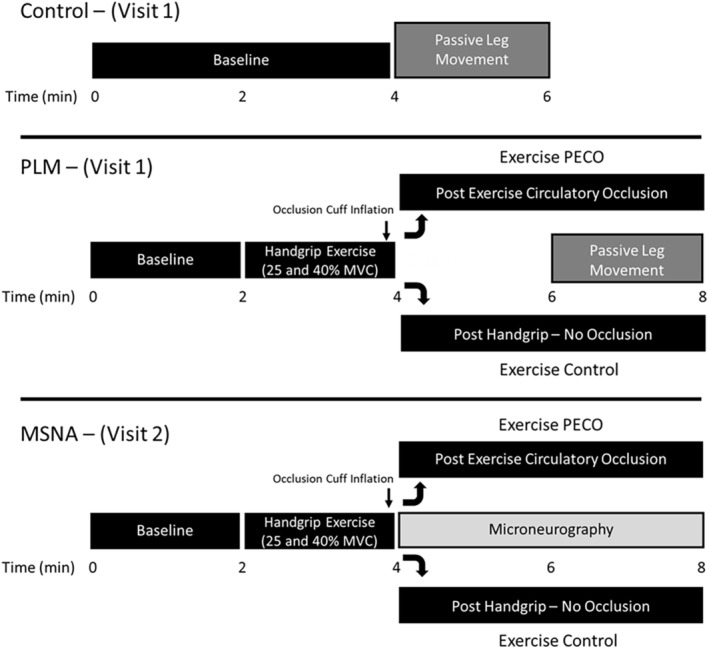
Experimental protocol consisting of two study visits. Visit 1: Five passive leg movement (PLM) trials including (1) CONTROL; PLM performed following 4 min of baseline, (2) ExCON 25%; PLM performed after 2 min of static isometric handgrip (HG) exercise at 25% MVC, (3) ExCON 40%; PLM performed after 2 min of static isometric HG exercise at 40% MVC, (4) ExPECO 25%; PLM performed after 2 min of static isometric HG exercise at 25% MVC with concomitant post exercise circulatory occlusion (PECO), and (5) ExPECO 40%; PLM performed after 2 min of static isometric HG exercise at 40% MVC with concomitant PECO. Visit 2: All trials, ExCON 25%, ExCON 40%, ExPECO 25%, and ExPECO 40% were performed followed by muscle sympathetic nerve activity (MSNA) without PLM, and recovery was reduced to 10 min between trials.

Subjects reported to the laboratory following an overnight fast (≥8 h) on two separate occasions. The experimental protocol was separated into two visits; one visit focusing on the assessment of central and peripheral hemodynamics during static isometric handgrip (HG) exercise and subsequent PLM (as described below) with and without PECO, while the second visit focused on recording MSNA following HG exercise and PECO without PLM (Figure 1). We chose to perform two study visits due to the extended duration of the PLM visit (3–4 h) and the difficulty in maintaining an adequate MSNA signal during the PLM, as leg movement during the assessment can interfere with the MSNA measurement. Successful recordings of MSNA were obtained in eight of the 11 subjects.

Upon arrival at the laboratory, body mass and height were recorded and subjects were instrumented while in the seated upright position. Following instrumentation, subjects performed a series of 3 maximal voluntary handgrip contractions (MVC; 47 ± 3 kg) separated by 1 min recovery. Additionally, subjects were familiarized with the PLM assessment (described below) to ensure avoidance of voluntary contraction during the passive movement test. Following 10 min of recovery from familiarization, and attainment of stable central and hemodynamic measures, the experimental trials commenced. During the PLM visit (visit 1), a total of five experimental trials were performed as depicted in the protocol timeline (Figure 1). Subjects completed a single control PLM with no intervention and 4 PLMs following the HG intervention. Each trial included 2 min of baseline followed by 2 min of static isometric HG exercise at 25% or 40% MVC. Immediately prior to the end of the 2 min HG exercise, an occlusion cuff placed proximal to the elbow was inflated to suprasystolic pressure (250 mmHg) (ExPECO) or left deflated for control trials (ExCON). PECO isolates the contribution of the metaboreceptors to the exercise pressor reflex resulting in elevated mean arterial pressure (MAP) and MSNA during cuff occlusion (Alam & Smirk, 1937; Boushel, 2010). Two min into the ExPECO or ExCON condition, PLM commenced following established guidelines (Gifford & Richardson, 2017). Briefly, while subjects sat in the upright position with the right leg fully extended (i.e., 180°), a member of the research team moved the leg through a 90° range of motion (i.e., flexion and extension) repeatedly at a rate of 1 Hz for 2 min. Subjects were instructed to remain passive and resist the urge to assist with leg movement. A 20 min period of recovery separated trials ensuring stable central and peripheral hemodynamics before the start of the next trial. The order of the trials was balanced such that participants were assigned to conditions in a way that evenly distributed the order of each condition across all participants. Additionally, the order of the PECO and CONTROL trials were kept consistent within each participant.

During the MSNA visit (visit 2) subjects were positioned in the upright seated position and MSNA was continuously recorded from the peroneal nerve. The protocol described above, including handgrip exercise, was repeated; however, peripheral hemodynamics were not assessed and PLM was not performed. Additionally, due to concern with maintaining an adequate MSNA signal the recovery between trials was reduced to 10 min. Prior to the start of each trial, MAP was assessed to ensure similar levels between trials.

### 2.3 Experimental measurements

#### 2.3.1 Leg blood flow

Common femoral artery blood velocity and vessel diameter were measured in the passively moved limb distal to the inguinal ligament and proximal to the deep and superficial femoral bifurcation with a Logic 7 Doppler ultrasound system (General Electric Medical Systems, Milwaukee, WI, USA) according to published guidelines (Limberg et al., 2020). The ultrasound system was equipped with a linear transducer operating at an imaging frequency of 10 MHz. Blood velocity was measured using the same transducers with a frequency of 5 MHz. All blood velocity measurements were obtained with the probes appropriately positioned to maintain an insonation angle of no more than 60°. Arterial diameter was measured, and mean velocity (*V*_*mean*_) (angle corrected, and intensity‐weighted area under the curve) was automatically calculated (Logic 7). Using arterial diameter and *V*_*mean*_, leg blood flow (LBF) in the femoral artery was calculated as *V*_*mean*_ × *π* × (vessel diameter/2)^2^ × 60, and reported as ml·min^−1^. Leg vascular conductance (LVC), an index of vasodilation, was calculated as LBF·MAP^−1^ and expressed as ml·min^−1^ mmHg^−1^.

#### 2.3.2 Central hemodynamics

Heart rate was monitored using a 3 lead electrocardiogram (Biopac). Beat‐by‐beat changes in stroke volume (SV), cardiac output (CO), and mean arterial pressure (MAP) were continuously determined by a finger photoplethysmography (Finometer: Finapres Medical Systems, Amsterdam, the Netherlands). Additionally, systolic blood pressure (SBP) and diastolic blood pressure (DBP) were determined by auscultation of the left arm with use of an automated sphygmomanometer (Tango^+^, Suntech, Morrisville, NC) to verify the Finometer readings at min 1, 3, 5, and 7 of each experimental trial. MAP was calculated as DBP + 1/3 (SBP−DBP).

#### 2.3.3 MSNA

**FIGURE 2:**
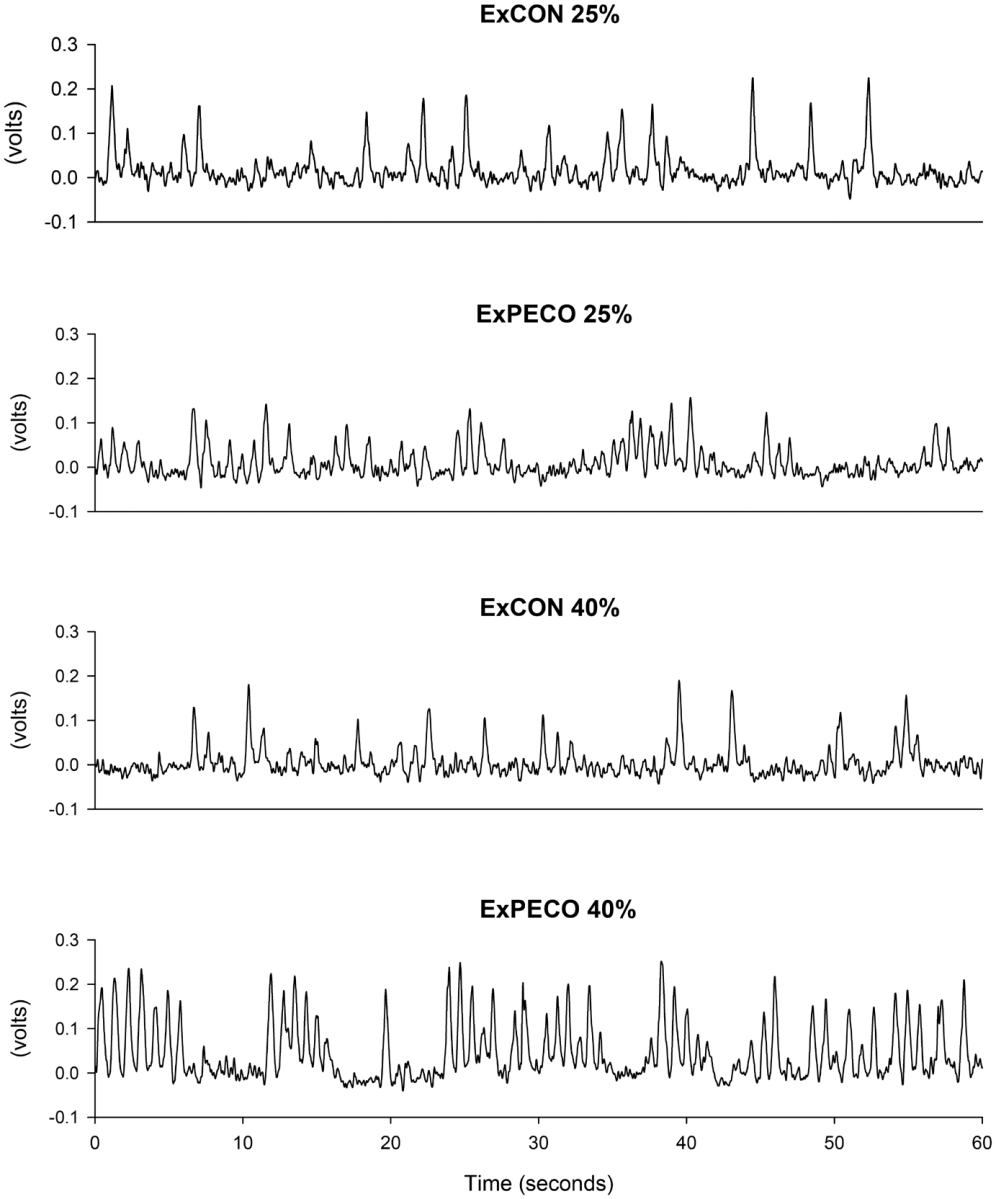
Muscle sympathetic nerve activity (MSNA) following handgrip exercise performed with (ExPECO 25%, ExPECO 40%) and without (ExCON 25%, ExCON 40%) post exercise circulatory occlusion (PECO) in a representative subject.

Multiunit postganglionic MSNA was recorded using standard microneurographic techniques as previously described (Sundlof & Wallin, 1978). Following careful mapping of the peroneal nerve near the fibular head on the right leg, a unipolar tungsten microelectrode was inserted into the muscle fascicle. Neural signals were processed by a preamplifier (Nerve traffic analyzer model 662C‐3, Iowa Bioengineering, Iowa City, IA, USA) with a total gain of 90,000. Amplified signals were filtered (bandwidth 700–2000 Hz), rectified, and integrated (time constant 0.1 s) to obtain mean voltage neurograms. Representative tracing of MSNA during ExPECO and ExCON is presented in Figure 2. Pulse‐synchronous MSNA bursts were identified manually by an experienced microneurographer according to appearance (3:1 ratio above the background noise) and timing in relation to the preceding ECG R‐wave. Burst frequency (burst·min^−1^) and burst incidence (burst·100 beats^−1^) are reported.

#### 2.3.4 Data acquisition

Throughout each protocol, HR, SV, CO, MAP, and ECG signals underwent analog‐to‐digital conversion and were simultaneously acquired (200 Hz) using a data acquisition system, (AcqKnowledge; Biopac Systems, Goleta, CA, USA).

#### 2.3.5 Data and statistical analysis

The data acquisition software allowed second‐by‐second analyses of HR, SV, CO, and MAP. All analyses were performed using a 5 s moving average. Second‐by‐second blood velocities were analyzed on the ultrasound system (GE Logic 7) for the first 60 s of movement, data from 60 s to 120 s were not included in the statistical analysis as the hyperemic response returned to baseline measures by the 60 s time point. Outcomes related to PLM including peak, delta (∆) peak, and total (area under the curve, AUC) FBF and FVC, as well as hemodynamic measures and MSNA were assessed via two‐way and one‐way repeated‐measures analysis of variance to determine significant differences between conditions. When a significant main effect (interaction of treatment by time) was observed, further post hoc analysis (Holm‐Sidak) was performed to determine significant differences between conditions. Significance was set a priori at *α* level of <0.05, and data are presented as means ± SD. A post hoc power analysis indicated a power of 0.98, suggesting adequate power for the given sample size.

## 3 RESULTS

### 3.1 Central and peripheral hemodynamics

#### 3.1.1 PRE exercise

**FIGURE 3:**
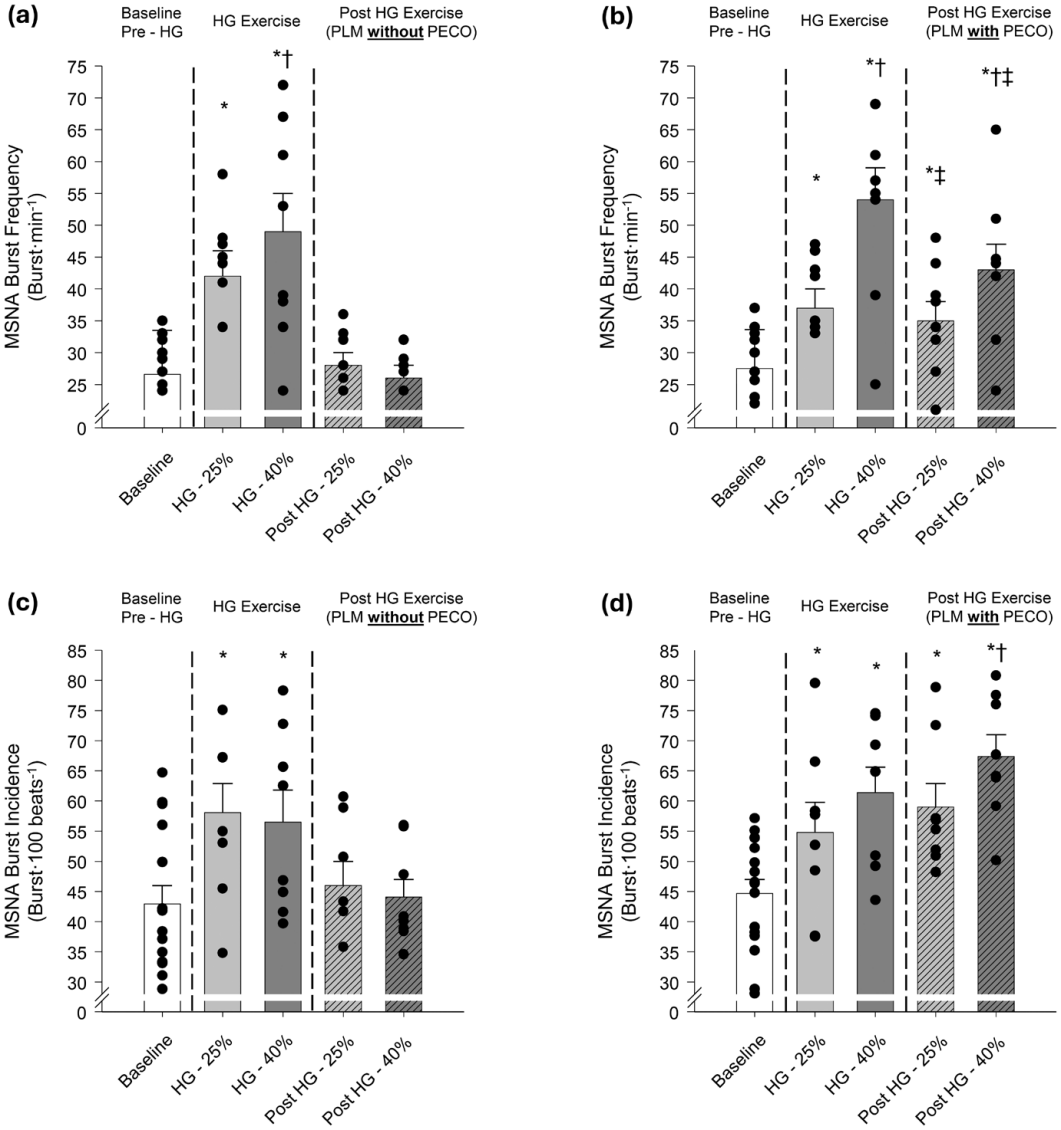
(a) Muscle sympathetic nerve activity (MSNA) burst frequency (burst·min^−1^) prior to, during, and post handgrip exercise *without* post exercise circulatory occlusion (PECO) and (b) *with* PECO and (c) MSNA burst incidence (burst·100 beats^−1^) *without* PECO and (d) *with* PECO. * Significant difference from CONTROL baseline, † significant difference between exercise intensities, ‡ significant difference from same exercise intensity during handgrip exercise, *p* < 0.05.

Prior to HG exercise, baseline central hemodynamics (HR, SV, and CO) were generally similar between conditions with the only exception of a slight (~3 bpm) elevation in HR prior to ExCON 40% and ExPECO 40% when compared to CONTROL (Table 1). MAP was slightly higher at baseline for ExCON 40% than ExCON 25%. There were no baseline differences in HR, SV, CO, or MAP when comparing ExCON 25% to ExPECO 25% or ExCON 40% to ExPECO 40%. Baseline LBF and LVC were not different between any of the conditions (Table 1). Measures of MSNA burst frequency and burst incidence were not different between conditions (Figure 3).

**TABLE 1:** Central and peripheral hemodynamics before, during, and after handgrip exercise.

	CONTROL	ExCON 25%	ExPECO 25%	ExCON 40%	ExPECO 40%
Heart rate, bpm					
Pre HG baseline	59 ± 9	61 ± 9	61 ± 10	62 ± 8^a^	62 ± 4^a^
HG exercise		73 ± 14^b^	74 ± 14^b^	86 ± 15^b^, ^c^	89 ± 15^b^, ^c^
Post HG exercise		59 ± 10	60 ± 11	58 ± 10	65 ± 17^e^
Stroke volume, mL·beat^−1^					
Pre HG baseline	93 ± 8	84 ± 21	94 ± 18	90 ± 13	92 ± 12
HG exercise		92 ± 15	94 ± 17	92 ± 24	87 ± 14
Post HG exercise		102 ± 13^b^	105 ± 18^b^	105 ± 15^b^	104 ± 10^b^
Cardiac output, L·min^−1^					
Pre HG baseline	5.2 ± 0.8	5.4 ± 0.7	5.5 ± 1.0	5.5 ± 0.7	5.7 ± 1.1
HG exercise		6.7 ± 1.6^b^	6.6 ± 2.3^b^	8.1 ± 2.7^b^, ^c^	7.9 ± 1.4^b^, ^c^
Post HG exercise		5.9 ± 0.8	6.2 ± 1.3	6.0 ± 0.9	6.6 ± 1.4
Mean arterial pressure, mmHg					
Pre HG baseline	87 ± 8	86 ± 6	86 ± 8	92 ± 7^c^	89 ± 10
HG exercise		118 ± 17^b^	119 ± 15^b^	138 ± 21^b^, ^c^	139 ± 22^b^, ^c^
Post HG exercise		88 ± 9	108 ± 15^d^	90 ± 12	119 ± 13^d^
Leg blood flow, mL·min^−1^					
Pre HG baseline	418 ± 147	469 ± 229	465 ± 223	479 ± 192	520 ± 227
HG exercise		677 ± 476^b^	681 ± 464^b^	733 ± 411^b^	730 ± 425^b^
Post HG exercise		538 ± 368	640 ± 336	516 ± 281	700 ± 488^d^
Leg vascular conductance, mL·min^−1^·mmHg^−1^					
Pre HG baseline	5.0 ± 2.0	5.6 ± 2.8	5.6 ± 3.1	5.3 ± 2.4	6.1 ± 3.6
HG exercise		5.8 ± 3.8	6.0 ± 4.5	5.3 ± 2.9	5.4 ± 3.3
Post HG exercise		6.3 ± 4.4	6.0 ± 3.3	6.0 ± 3.8	6.0 ± 4.4

*Note*: Values are means ± SD for 11 males. Data were analyzed with two‐way repeated‐measures ANOVA, followed by Holm‐Sidak post hoc test (when a significant interaction was detected).

^a^ Significant difference from CONTROL.

^b^ Significant difference from baseline within trial.

^c^ Significant difference between exercise intensities (25% vs. 40%).

^d^ Significant difference between ExCON and ExPECO at the same exercise intensity.

^e^ Significant difference from all other conditions, *p* < 0.05.

#### 3.1.2 During exercise

Isometric HG exercise at 25% and 40% of MVC evoked graded elevations in HR, CO, MAP (Table 1), and MSNA burst frequency (Figure 3a,b). MSNA burst incidence was elevated during HG exercise with no differences between ExPECO and ExCON conditions (Figure 3c,d). LBF was augmented by HG exercise, however, no differences were present between conditions. LVC remained unchanged during HG exercise (Table 1).

#### 3.1.3 Post exercise

Following HG exercise, HR and CO returned to pre‐HG levels while SV was uniformly increased across conditions (Table 1). HR was elevated during ExPECO 40% compared to all other conditions. MAP and MSNA returned to pre‐HG levels after cessation of HG exercise during ExCON 25% and ExCON 40% (Tables 1, 2, and Figure 3) indicating that sympathetic activation returned to baseline levels. Conversely, engaging the metaboreflex after HG exercise resulted in elevated MAP and MSNA during ExPECO 25% and ExPECO 40% (Tables 1, 2, and Figure 3). Importantly, the metaboreflex‐induced increases in MAP and MSNA were graded with respect to the intensity of the preceding HG exercise (i.e., ExPECO 40% > ExPECO 25% > CONTROL). Following HG exercise, LBF returned to pre‐HG levels during ExCON 25% and ExCON 40% and remained elevated during both ExPECO 25% and ExPECO 40%. LVC remained unchanged following HG exercise.

**TABLE 2:** Hemodynamic responses during passive limb movement.

	CONTROL	ExCON 25%	ExPECO 25%	ExCON 40%	ExPECO 40%
Heart rate, BPM					
Pre PLM baseline	59 ± 9	59 ± 10	60 ± 11	58 ± 10	65 ± 17^d^
Peak	63 ± 8^a^	66 ± 11^a^	69 ± 11^a^	63 ± 9^a^	71 ± 18^a^
∆ Peak	4 ± 3	7 ± 6	9 ± 7	5 ± 4	6 ± 5
Stroke volume, mL·beat^−1^					
Pre PLM baseline	93 ± 8	102 ± 13	105 ± 18	105 ± 15	104 ± 10
Peak	102 ± 10^a^	110 ± 11^a^	116 ± 20^a^	114 ± 17^a^	115 ± 10^a^
∆ Peak	9 ± 5	8 ± 4	11 ± 5	9 ± 6	12 ± 8
Cardiac output, L·min^−1^					
Pre PLM baseline	5.2 ± 0.8	5.9 ± 0.8	6.2 ± 1.3^b^	6.0 ± 0.9	6.6 ± 1.4^b^
Peak	6.2 ± 1.3^a^	6.8 ± 0.8^a^	7.3 ± 1.6^a^	6.7 ± 0.9^a^	7.6 ± 1.6^a^, ^b^
∆ Peak	1.0 ± 0.8	0.9 ± 0.6	1.1 ± 1.0	0.7 ± 0.6	1.0 ± 1.4
Leg blood flow, mL·min^−1^					
Pre PLM baseline	418 ± 147	538 ± 368	640 ± 336^b^	516 ± 281	700 ± 488^b^
Peak	1229 ± 387^a^	1208 ± 576^a^	1380 ± 504^a^	1139 ± 313^a^	1258 ± 683^a^
∆ Peak	811 ± 295	670 ± 308	740 ± 391	623 ± 302	558 ± 377^b^
Total, AUC	264 ± 136	226 ± 131	252 ± 172	172 ± 133	216 ± 193
Leg vascular conductance, mL·min^−1^·mmHg^−1^					
Pre PLM baseline	4.9 ± 2.0	6.3 ± 4.4	6.0 ± 3.2	6.0 ± 3.8	6.0 ± 4.4
Peak	14.5 ± 5.6^a^	13.6 ± 6.8^a^	14.1 ± 5.8^a^	13.0 ± 3.8^a^	11.4 ± 6.4^a^, ^b^, ^c^
∆ Peak	9.6 ± 4.1	7.2 ± 3.4	8.1 ± 4.6	7.0 ± 3.9^b^	5.4 ± 3.7^b^, ^c^
Total, AUC	3.0 ± 1.8	2.5 ± 1.5	2.4 ± 1.6	1.8 ± 1.5	1.8 ± 1.7^b^

*Note*: Values are means ± SD for 11 males. Data were analyzed with two‐way repeated‐measures ANOVA, followed by Holm‐Sidak post hoc test (when a significant interaction was detected).

^a^ Significant difference from baseline.

^b^ Significant difference from CONTROL.

^c^ Significant difference from PECO 25%.

^d^ Significant difference from all other trials, *p* < 0.05.

### 3.2 Central and peripheral hemodynamics during PLM

**FIGURE 4:**
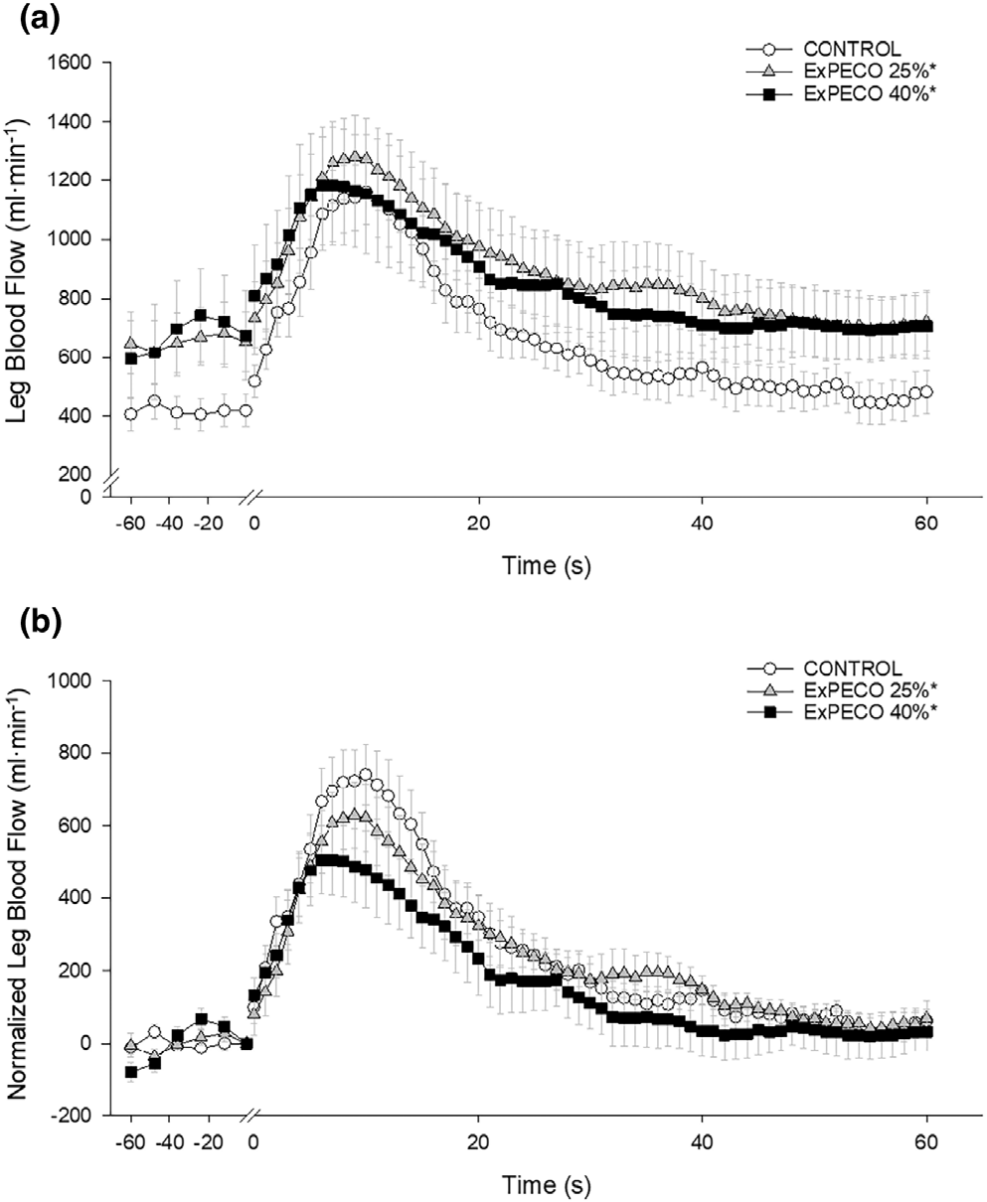
(a) Absolute leg blood flow (LBF, mL·min^−1^) and (b) normalized LBF (baseline normalized to 0, mL·min^−1^) during passive leg movement with PECO. Passive leg movement started at time 0 and was performed continuously for 60 s. * Significant difference for the overall LBF response from CONTROL, *p* < 0.05.

**FIGURE 5:**
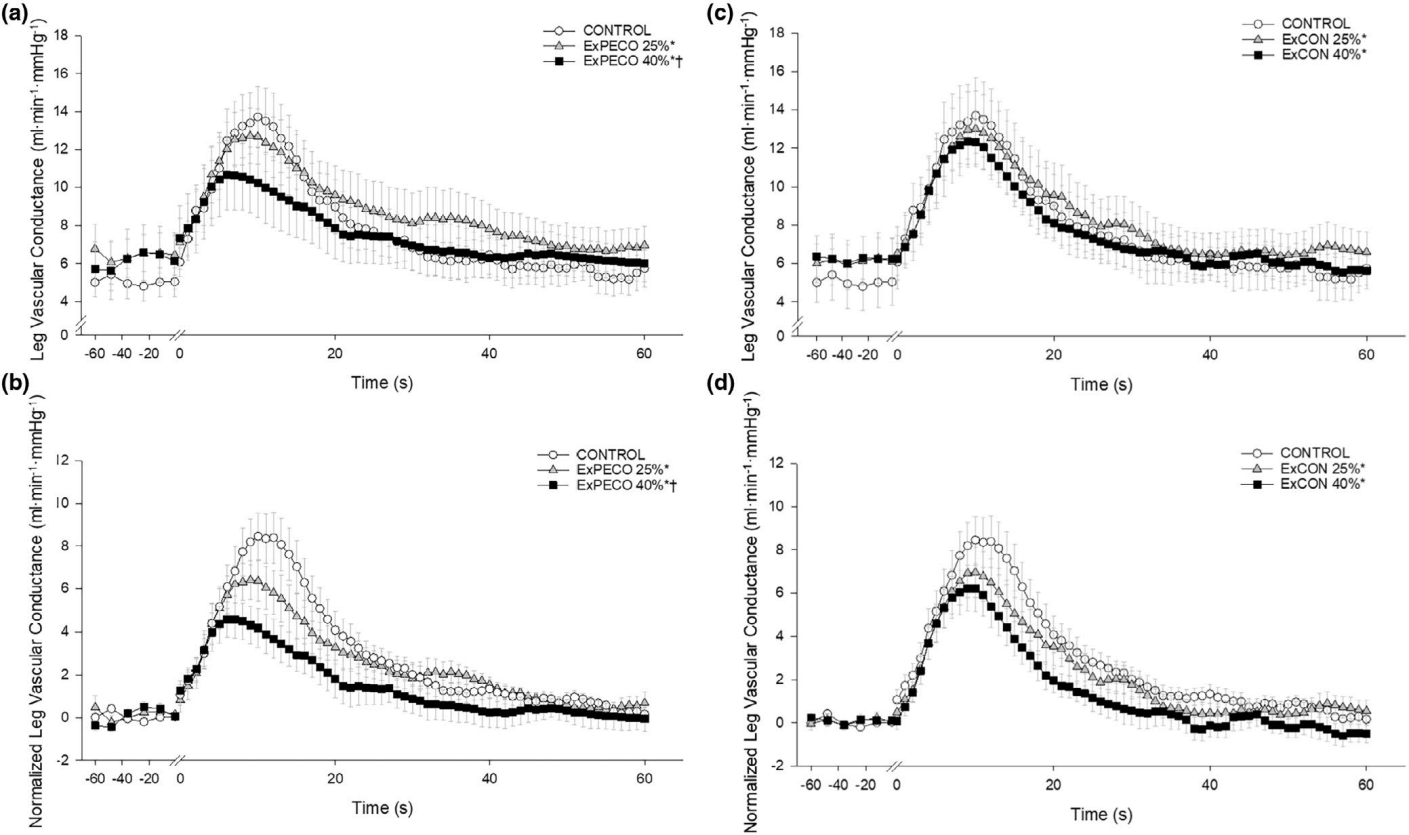
(a) Absolute leg vascular conductance (LVC, mL·min^−1^·mmHg^−1^) and (b) normalized LVC (baseline normalized to 0, mL·min^−1^·mmHg^−1^) during passive leg movement with PECO; and (c) absolute leg vascular conductance (LVC, mL·min^−1^·mmHg^−1^) and (d) normalized LVC (baseline normalized to 0, ml·min^−1^·mmHg^−1^) during passive leg movement without PECO. Passive leg movement started at time 0 and was performed continuously for 60 s. * Significant difference for the overall LVC response from CONTROL, † significant difference from PECO 25%, *p* < 0.05.

PLM‐induced a robust and transient increase in LBF (Figure 4a) and LVC (Figure 5). During CONTROL, LBF and LVC increased nearly 3‐fold. Significant differences existed between PLM‐induced LBF when comparing CONTROL to ExPECO 25% and ExPECO 40% (Figure 4a). These differences are largely accounted for by the augmented baseline LBF prior to PLM (Table 2 and Figure 4a). Normalizing for the elevated baseline LBF revealed that the hyperemic response was unaltered by ExPECO 25% but reduced during ExPECO 40% (Figure 4b).

**FIGURE 6:**
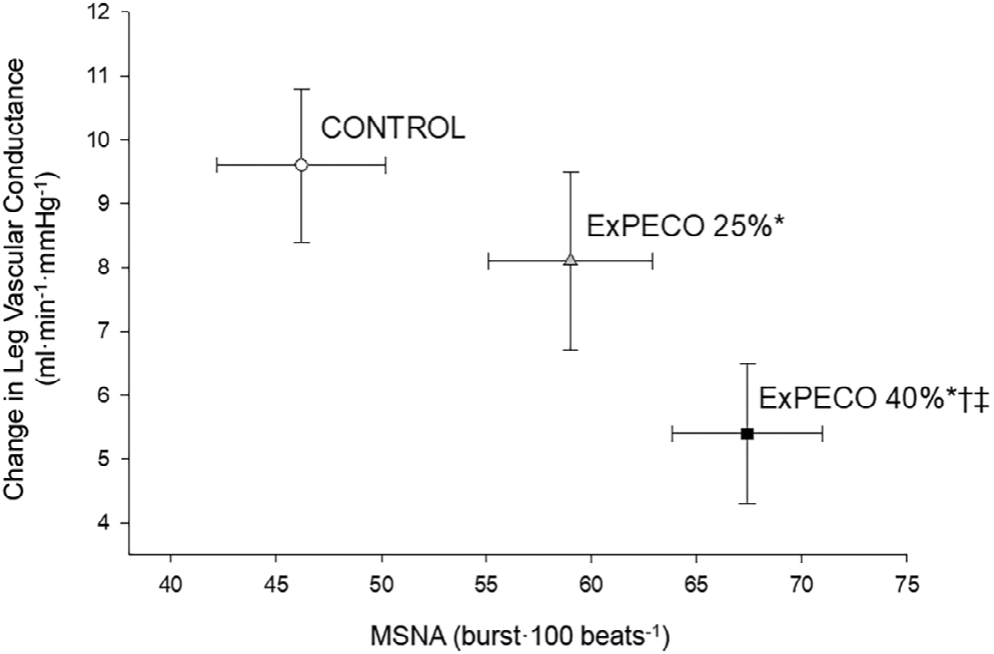
PLM‐induced change in LVC (Δ ml·min^−1^·mmHg^−1^) verse MSNA (burst·100 beats^−1^). Graded increases in MSNA evoked by PECO and corresponding reductions in the change in LVC. * Significant difference in MSNA from CONTROL, † significant difference in MSNA from ExPECO 25%, ‡ significant difference in LVC from CONTROL and ExPECO 25%, *p* < 0.05.

During ExPECO 25% and ExPECO 40% the vasodilatory response to PLM was incrementally reduced compared to CONTROL (Figures 5 and 6). Interestingly, when PLM was preceded by HG exercise alone, a modest, albeit significant reduction in the vasodilatory response to PLM was observed during both ExCON 25% and ExCON 40% (Figure 5). Unlike the PECO conditions, this reduction in the vasodilatory response was not dependent on the intensity of the preceding HG exercise bout, as the reduction in LVC during ExCON 25% and ExCON 40% was similar. Accounting for this unexpected impact of HG on the PLM response revealed that the PLM‐induced vasodilatory response was not different between ExCON 25% and ExPECO 25% but was reduced during ExPECO 40% when compared to all other conditions. Additionally, peak and total LVC during ExPECO 40% was attenuated compared to CONTROL and ExPECO 25% (Table 2).

PLM evoked increases in HR, SV, and CO that were not different between conditions (Table 2). Peak HR and SV were not different between conditions while peak CO during ExPECO 40% was higher than CONTROL (Table 2).

## 4 DISCUSSION

Vascular dysfunction and impaired vasodilation often occur in parallel with heightened MSNA, which is associated with aging and the development and progression of cardiovascular disease. However, how acute sympathoexcitation impacts the vasodilatory response to PLM, an assessment of vascular function, has not been determined independent of systemic cardiovascular responses that occur during exercise (Venturelli et al., 2022). The novel findings from this study indicate that acute sympathetic activation, accomplished by post exercise circulatory occlusion (PECO), diminishes the hyperemic and vasodilatory responses to PLM, suggesting a regulatory influence of sympathetic nervous system activation on microvascular function in young, healthy males.

### 4.1 Impact of acute sympathoexcitation on the vasodilatory response to PLM

PLM provides a primarily NO‐dependent assessment of vascular function (Mortensen et al., 2012; Trinity et al., 2012, 2021). Conditions associated with vascular dysfunction including aging, heart failure, and spinal cord injury exhibit reductions in PLM‐induced vasodilation (Hayman et al., 2010; McDaniel, Hayman, et al., 2010). In the current study, acute elevations in MSNA directly impaired the vasodilatory response to PLM. Specifically, during metaboreceptor activation following HG exercise at 25% and 40% MVC (i.e., ExPECO 25% and ExPECO 40%) the change in LVC was reduced by 16% and 44%, respectively, when compared to CONTROL (Figure 5 and Table 2). It is important to note that the reduction in LVC in the ExPECO 25% group falls within the between day coefficient of variation (CV) of ~10%–25% previously described for PLM (Gifford & Richardson, 2017; Groot et al., 2022). Regardless, the significant reductions in LVC coincided with significant increases in MSNA in both ExPECO 25% and ExPECO 40% (Figure 6). Moreover, the effect size (Cohen's D) for the reduction in LVC during ExPECO 25% and ExPECO 40% were 0.35 and 0.70, respectively. These findings support recent findings from our group of stepwise reductions in PLM‐induced vasodilation during progressive exercise‐mediated increases in MSNA (Venturelli et al., 2022) and extend these findings to local activation of this reflex loop by the metaboreceptors. The concomitant elevation in MSNA and reduction in the hyperemic response to PLM helps to elucidate factors in addition to diminished NO bioavailability, such as altered mechanoreflex sensitivity (Trinity et al., 2010; Witman et al., 2015), that may contribute to previous reports of reduced PLM‐induced vasodilation with aging and heart disease (Groot et al., 2013; McDaniel, Hayman, et al., 2010; Trinity et al., 2015).

The mechanisms contributing to the reduced vasodilation during elevated sympathetic activation are not entirely clear. During PECO, MSNA increases systemically in response to localized metaboreceptor activation (Hansen et al., 1994). This global increase in sympathetic outflow and stimulus for vasoconstriction may directly oppose the vasodilatory response to PLM by some general, yet unrecognized, mechanism. Conversely, elevated sympathetic outflow may directly inhibit NO. Hijmering et al. (2002) reported a reduction in FMD following acute sympathetic activation (via lower body negative pressure) and attributed the reduction in FMD to a specific inhibitory effect of sympathetic activation on shear‐mediated NO release. In vitro evidence suggests that norepinephrine may lead to inactivation of NO (Rubanyi et al., 1985) lending support to this potential mechanism, however, evidence of this occurring in vivo is lacking (Rector et al., 1993). A third potential mechanism may involve an imbalance between NO bioavailability and MSNA. Physiologically, NO and MSNA are antagonistic, as NO promotes vasodilation whereas increased MSNA evokes vasoconstriction. Therefore, increasing MSNA without an increase in NO (as would be expected given the peak hyperemic response was not altered by PECO (Table 2)), would shift the balance toward vasoconstriction and reduction in LVC during PLM. Both animal and human models demonstrate an interaction between NO and α‐adrenergic function such that NO acts to attenuate α‐adrenergic vasoconstriction (Dietz et al., 1997; Fadel et al., 2012), however, how such a mechanism may manifest in humans during PLM is not certain. Based on the current findings, we are unable to determine if the reduction in LVC during PLM is due to a general increase in sympathetically‐mediated vasoconstriction, a direct impact on NO, or a combination of these aforementioned mechanisms.

The control trials (i.e., ExCON 25% and ExCON 40%), yielded unexpected reductions in the vasodilatory response to PLM that were not associated with heightened MSNA (Figure 5). At the cessation of HG exercise, MSNA and MAP returned to pre‐HG levels indicating that sympathetic activation likely does not account for the observed reductions in LVC (Figure 3). Alternatively, the reduced LVC during PLM may be explained by the increase in LBF during HG exercise, which may have resulted in activation of endothelial nitric oxide synthase (eNOS) and a subsequent increase in NO bioavailability (Casey et al., 2017). Increasing LBF prior to PLM with heating effectively reduced the PLM‐induced hyperemic response (Shields et al., 2021). Recent human and animal work suggests that vascular responsiveness, as measured by vasodilation, is reduced with repeated stimulation due to potential “resetting” of the endothelium (Turturici & Roatta, 2013) or alterations in tissue oxygenation (Messere et al., 2017). In keeping with this notion, activation of eNOS prior to PLM may have decreased NO bioavailability leading to the observed and marked reduction in LVC at the initiation of PLM. Importantly, this does not negate our finding that MSNA plays a significant role in the LVC response as the increase in LBF during and after HG was nearly identical between ExPECO 25% and ExPECO 40%, yet LVC was reduced during ExPECO 40% when compared to ExPECO 25%.

The impact of acute sympathoexcitation on vascular function, assessed by FMD, has been examined previously during lower body negative pressure, mental stress, cold pressor test, and metaboreceptor engagement (Dyson et al., 2006; Hijmering et al., 2002; Lind et al., 2002; Thijssen et al., 2006). The findings of these investigations are equivocal, as FMD has been reported to be unchanged, improved, or impaired due to increased sympathetic activity. Reductions in FMD ranging from 40% to 60% have been reported during sympathetic activation induced by lower body negative pressure and cold pressor test (Hijmering et al., 2002; Lind et al., 2002). Conversely, metaboreceptor activation reportedly increased FMD by nearly 2‐fold (Dyson et al., 2006). The variability in the FMD response to acute sympathetic activation in these previous investigations is likely attributed to disparate techniques used to evoke sympathetic activation, as well as uncertainty regarding the magnitude of changes in MSNA. Moreover, uncertainty with regard to the mechanisms contributing to vasodilation during FMD may further confound interpretation of these findings (Pyke et al., 2010; Wray et al., 2013). Thus, while differences in methodology between these former studies and the current work preclude a direct comparison, the present findings provide new evidence for the capacity of the sympathetic nervous system to diminish lower limb hyperemic and vasodilatory responsiveness in young, healthy males. It should also be noted that the present findings are specific to microvascular function, whereas the results of the aforementioned studies center around the FMD assessment of macrovascular function.

### 4.2 Regulation of hyperemia during PLM: Impact of acute sympathoexcitation

The unique experimental design employed in the present study provided the opportunity to assess the impact of sympathoexcitation on microvascular reactivity without the influence of metabolic vasodilation that accompanies voluntary exercise (Richardson et al., 1993; Saltin et al., 1998). This reductionist approach is particularly beneficial considering the complexity of neurovascular control during exercise, when sympathetic vasoconstriction to active skeletal muscle is diminished in direct relation to the exercise intensity, a phenomenon termed functional sympatholysis (Remensnyder et al., 1962; Saltin & Mortensen, 2012). Thus, the present investigation extends previous investigations examining the impact of augmented MSNA on skeletal muscle blood flow regulation during voluntary exercise (Joyner et al., 1990, 1992; Sinoway & Prophet, 1990; Strange, 1999), offering new insight regarding the capacity of sympathetic nervous system activity to regulate muscle blood flow in the resting (inactive) and passively moved limbs.

Prior to PLM, graded activation of the metaboreflex progressively increased MAP, which translated to elevated LBF and unchanged LVC (Table 2) despite a large and systemic increase in sympathetic outflow. This finding, which agrees with several previous reports in humans (Kilbom & Brundin, 1976; Strange, 1999), suggests that a systemic increase in sympathetic outflow does not elicit vasoconstriction in resting, inactive skeletal muscle. Without a vasodilatory stimulus such as PLM, the impact of metaboreceptor activation is unappreciated as LBF is increased yet LVC is unaltered. The full impact of metaboreflex activation and concomitant elevation in sympathetic nervous system activity is only realized after the initiation of PLM to stimulate blood flow. Indeed, PLM evoked a nearly 3‐fold increase in hyperemia during CONTROL (Figure 3). Peak hyperemia remained relatively unchanged despite marked and graded elevations in MSNA and MAP during ExPECO 25% and ExPECO 40% (Table 2). However, the overall vasodilatory response was attenuated during ExPECO 25% and to a greater effect during ExPECO 40% when compared to CONTROL (Figure 5). The reduction in LBF and LVC during PLM provide novel evidence of vasoconstriction even in the “active” (i.e., passively moved leg) muscle during heightened sympathetic activity.

### 4.3 Central hemodynamic contribution to PLM

The peripheral hemodynamic response to PLM is primarily governed by peripheral factors, however, central factors (HR, SV, and CO) do contribute to alterations reported with body position, age, and cardiovascular disease (Hayman et al., 2010; McDaniel, Hayman, et al., 2010; Trinity et al., 2010, 2011). In the current study, HG exercise elicited increases in HR, CO, and MAP. In general, these variables returned to pre‐HG levels following termination of HG exercise. By design, the increases in MAP and MSNA remained elevated during ExPECO, albeit at a slightly lower level than HG exercise, confirming the important role of central command in regulating cardiovascular responses to voluntary exercise (Mitchell et al., 1989; Strange, 1999; Victor et al., 1989). Despite modest differences in central hemodynamics prior to PLM, the changes induced by PLM were similar between conditions suggesting an important and consistent central hemodynamic contribution to PLM. Overall, the reductions in LBF and LVC during the PECO conditions do not appear to be due to altered central hemodynamics.

### 4.4 Experimental considerations

The current findings are novel and significant; however, only male subjects were evaluated. Although previous investigations have reported no sex‐related differences in the hyperemic and vasodilatory response to PLM (Weggen et al., 2023), young males express greater MSNA compared to their female counterparts (Briant et al., 2016; Hart et al., 2009). Therefore, future investigations including females are warranted to investigate how potential basal differences in MSNA between sexes may impact the hyperemic and vasodilatory response to PLM. Similarly, the current study did not include older adults who typically have elevated resting MSNA and impaired PLM (Trinity et al., 2015). Previously, using a pharmacological approach, we reported that elevated α‐adrenergic tone does not contribute to the attenuated PLM response in older adults (Fermoyle et al., 2023). Therefore, it is possible that similar findings may be observed with the current study design when applied to older adults; however, future investigation is needed to clarify the role of sympathetic activity on PLM in older adults. An additional limitation is the lack of MSNA measures during the PLM assessment. Measuring PLM and MSNA on separate visits was done due to the difficulty of obtaining and maintaining an adequate MSNA signal during the PLM assessment. Measurements of MSNA during PLM would provide a more accurate depiction of the relation between PLM and elevated MSNA. However, it is not expected that PLM is a sympathoexcitatory maneuver and thus would not likely increase MSNA measures any further. It should also be noted that the day‐to‐day variability of sympathetic activity and the MSNA measure itself may also limit the interpretation of these data. Finally, the current study does not directly investigate any direct mechanistic relation between NO and sympathetic outflow. Unfortunately, the eNOS inhibitor *N* ^G^‐monomethyl‐l‐arginine is not currently available for human administration, thus, any insight into this mechanism is limited to animal models. Future investigations into this mechanistic interplay, certainly in appropriate animal models, can greatly add to our understanding of this connection.

## 5 CONCLUSION

In summary, heightened MSNA is associated with a negative impact on the vasodilatory and hyperemic responses to PLM in young males. Additionally, PLM provides a unique approach to further understand the impact of heightened sympathetic activity on blood flow regulation without the confounding influence of local metabolic vasodilation and central command that occur with voluntary exercise. These findings add to our understanding of sympathetic control of the vasculature and underscore the importance of accounting for differences in sympathetic activity when examining vascular function by PLM, at least within the understanding of young male adults.

## AUTHOR CONTRIBUTIONS

J.D.T. and D.W.W. conceived and designed research; J.F.L., R.S.G., Z.B.‐O., G.L., D.W.W., R.S.R., and J.D.T. performed experiments; B.E.H. and J.D.T. analyzed data, interpreted results, prepared figures, and drafted manuscript; B.E.H., B.A.R., and J.D.T. edited and revised manuscript; B.E.H., J.F.L., R.S.G., Z.B.‐O., G.L., B.A.R., D.W.W., R.S.R., and J.D.T. approved final version of manuscript.

## FUNDING INFORMATION

This work was supported by the National Institutes of Health R01HL142603 (to J.D. Trinity), Department of Veterans Affairs Merit Award I01RX0003810 (to J.D. Trinity), Veterans Affairs Rehabilitation Research and Development Merit Awards E6910‐R, E1697‐R, E1572‐P, E2323‐R, and E3207‐R (to R.S. Richardson), VA Clinical Science Research and Development Career Development Award IK2CX002114 (to R.S. Richardson), and VA Senior Research Career Scientist Award E9275‐L (to R.S. Richardson).

## CONFLICT OF INTEREST STATEMENT

The authors report no conflicts of interest, financial, or otherwise.

## ETHICS STATEMENT

The Institutional Review Board of the University of Utah approved this study, and all procedures conformed to the Declaration of Helsinki.

## Data Availability

Data will be made available upon reasonable request.

## References

[phy270180-bib-0001] Alam, M. , & Smirk, F. H. (1937). Observations in man upon a blood pressure raising reflex arising from the voluntary muscles. The Journal of Physiology, 89(4), 372–383. 16994867 10.1113/jphysiol.1937.sp003485 PMC1395054

[phy270180-bib-0002] Boushel, R. (2010). Muscle metaboreflex control of the circulation during exercise. Acta Physiologica (Oxford, England), 199(4), 367–383. 10.1111/j.1748-1716.2010.02133.x 20353495

[phy270180-bib-0003] Briant, L. J. , Burchell, A. E. , Ratcliffe, L. E. , Charkoudian, N. , Nightingale, A. K. , Paton, J. F. , Joyner, M. J. , & Hart, E. C. (2016). Quantifying sympathetic neuro‐haemodynamic transduction at rest in humans: Insights into sex, ageing and blood pressure control. The Journal of Physiology, 594(17), 4753–4768. 10.1113/jp272167 27068560 PMC5009776

[phy270180-bib-0004] Casey, D. P. , Ueda, K. , Wegman‐Points, L. , & Pierce, G. L. (2017). Muscle contraction induced arterial shear stress increases endothelial nitric oxide synthase phosphorylation in humans. American Journal of Physiology. Heart and Circulatory Physiology, 313(4), H854–H859. 10.1152/ajpheart.00282.2017 28801524 PMC5668602

[phy270180-bib-0005] Charkoudian, N. , & Rabbitts, J. A. (2009). Sympathetic neural mechanisms in human cardiovascular health and disease. Mayo Clinic Proceedings, 84(9), 822–830. 19720780 10.4065/84.9.822 PMC2735432

[phy270180-bib-0006] DeLorey, D. S. (2021). Sympathetic vasoconstriction in skeletal muscle: Modulatory effects of aging, exercise training, and sex. Applied Physiology, Nutrition, and Metabolism, 46(12), 1437–1447. 10.1139/apnm-2021-0399 34348066

[phy270180-bib-0007] Dietz, N. M. , Engelke, K. A. , Samuel, T. T. , Fix, R. T. , & Joyner, M. J. (1997). Evidence for nitric oxide‐mediated sympathetic forearm vasodiolatation in humans. The Journal of Physiology, 498(Pt_2), 531–540. 9032700 10.1113/jphysiol.1997.sp021879 PMC1159222

[phy270180-bib-0008] Dinenno, F. A. , Jones, P. P. , Seals, D. R. , & Tanaka, H. (2000). Age‐associated arterial wall thickening is related to elevations in sympathetic activity in healthy humans. American Journal of Physiology. Heart and Circulatory Physiology, 278(4), H1205–H1210. 10749715 10.1152/ajpheart.2000.278.4.H1205

[phy270180-bib-0009] Dyson, K. S. , Shoemaker, J. K. , & Hughson, R. L. (2006). Effect of acute sympathetic nervous system activation on flow‐mediated dilation of brachial artery. American Journal of Physiology. Heart and Circulatory Physiology, 290(4), H1446–H1453. 10.1152/ajpheart.00771.2005 16284236

[phy270180-bib-0010] Fadel, P. J. , Farias Iii, M. , Gallagher, K. M. , Wang, Z. , & Thomas, G. D. (2012). Oxidative stress and enhanced sympathetic vasoconstriction in contracting muscles of nitrate‐tolerant rats and humans. The Journal of Physiology, 590(2), 395–407. 10.1113/jphysiol.2011.218917 22106180 PMC3285073

[phy270180-bib-0011] Fagius, J. , & Wallin, B. G. (1993). Long‐term variability and reproducibility of resting human muscle nerve sympathetic activity at rest, as reassessed after a decade. Clinical Autonomic Research, 3(3), 201–205. 8400820 10.1007/BF01826234

[phy270180-bib-0012] Fairfax, S. T. , Padilla, J. , Vianna, L. C. , Davis, M. J. , & Fadel, P. J. (2013). Spontaneous bursts of muscle sympathetic nerve activity decrease leg vascular conductance in resting humans. American Journal of Physiology. Heart and Circulatory Physiology, 304(5), H759–H766. 10.1152/ajpheart.00842.2012 23292718 PMC3602753

[phy270180-bib-0013] Fermoyle, C. C. , La Salle, D. T. , Alpenglow, J. K. , Craig, J. C. , Jarrett, C. L. , Broxterman, R. M. , McKenzie, A. I. , Morgan, D. E. , Birgenheier, N. M. , Wray, D. W. , Richardson, R. S. , & Trinity, J. D. (2023). Pharmacological modulation of adrenergic tone alters the vasodilatory response to passive leg movement in young but not in old adults. Journal of Applied Physiology (Bethesda, MD: 1985), 134(5), 1124–1134. 10.1152/japplphysiol.00682.2022 36927146 PMC10125034

[phy270180-bib-0014] Fisher, J. P. , Young, C. N. , & Fadel, P. J. (2015). Autonomic adjustments to exercise in humans. Comprehensive Physiology, 5(2), 475–512. 10.1002/cphy.c140022 25880502

[phy270180-bib-0015] Gifford, J. R. , & Richardson, R. S. (2017). CORP: Ultrasound assessment of vascular function with the passive leg movement technique. Journal of Applied Physiology (Bethesda, MD: 1985), 123(6), 1708–1720. 10.1152/japplphysiol.00557.2017 28883048 PMC5814681

[phy270180-bib-0016] Grassi, G. (1998). Role of the sympathetic nervous system in human hypertension. Journal of Hypertension, 16(12 Pt 2), 1979–1987. 9886886 10.1097/00004872-199816121-00019

[phy270180-bib-0017] Green, D. J. , Jones, H. , Thijssen, D. , Cable, N. T. , & Atkinson, G. (2011). Flow‐mediated dilation and cardiovascular event prediction: Does nitric oxide matter? Hypertension, 57(3), 363–369. 10.1161/hypertensionaha.110.167015 21263128

[phy270180-bib-0018] Groot, H. J. , Broxterman, R. M. , Gifford, J. R. , Garten, R. S. , Rossman, M. J. , Jarrett, C. L. , Kwon, O. S. , Hydren, J. R. , & Richardson, R. S. (2022). Reliability of the passive leg movement assessment of vascular function in men. Experimental Physiology, 107(5), 541–552. 10.1113/ep090312 35294784 PMC9058221

[phy270180-bib-0019] Groot, H. J. , Trinity, J. D. , Layec, G. , Rossman, M. J. , Ives, S. J. , & Richardson, R. S. (2013). Perfusion pressure and movement‐induced hyperemia: Evidence of limited vascular function and vasodilatory reserve with age. American Journal of Physiology. Heart and Circulatory Physiology, 304(4), H610–H619. 10.1152/ajpheart.00656.2012 23262136 PMC3566486

[phy270180-bib-0020] Hansen, J. , Thomas, G. D. , Jacobsen, T. N. , & Victor, R. G. (1994). Muscle metaboreflex triggers parallel sympathetic activation in exercising and resting human skeletal muscle. American Journal of Physiology. Heart and Circulatory Physiology, 266(6), H2508–H2514. 10.1152/ajpheart.1994.266.6.H2508 8024012

[phy270180-bib-0021] Hart, E. C. , Charkoudian, N. , Wallin, B. G. , Curry, T. B. , Eisenach, J. H. , & Joyner, M. J. (2009). Sex differences in sympathetic neural‐hemodynamic balance: Implications for human blood pressure regulation. Hypertension, 53(3), 571–576. 10.1161/hypertensionaha.108.126391 19171792 PMC3733790

[phy270180-bib-0022] Hayman, M. A. , Nativi, J. N. , Stehlik, J. , McDaniel, J. , Fjeldstad, A. S. , Ives, S. J. , Wray, D. W. , Bader, F. , Gilbert, E. M. , & Richardson, R. S. (2010). Understanding exercise‐induced hyperemia: Central and peripheral hemodynamic responses to passive limb movement in heart transplant recipients. American Journal of Physiology. Heart and Circulatory Physiology, 299, H1653–H1659. 10.1152/ajpheart.00580.2010 20833963 PMC2993203

[phy270180-bib-0023] Hijmering, M. L. , Stroes, E. S. G. , Olijhoek, J. , Hutten, B. A. , Blankestijn, P. J. , & Rabelink, T. J. (2002). Sympathetic activation markedly reduces endothelium‐dependent, flow‐mediated vasodilation. Journal of the American College of Cardiology, 39(4), 683–688. 10.1016/S0735-1097(01)01786-7 11849869

[phy270180-bib-0024] Joyner, M. J. , Lennon, R. L. , Wedel, D. J. , Rose, S. H. , & Shepherd, J. T. (1990). Blood flow to contracting human muscles: Influence of increased sympathetic activity. Journal of Applied Physiology, 68, 1453–1457. 2347787 10.1152/jappl.1990.68.4.1453

[phy270180-bib-0025] Joyner, M. J. , Nauss, L. A. , Warner, M. A. , & Warner, D. O. (1992). Sympathetic modulation of blood flow and O2 uptake in rhythmically contracting human forearm muscles. American Journal of Physiology. Heart and Circulatory Physiology, 263(4), H1078–H1083. 10.1152/ajpheart.1992.263.4.H1078 1415755

[phy270180-bib-0026] Kilbom, A. , & Brundin, T. (1976). Circulatory effects of isometric muscle contractions, performed separately and in combination with dynamic exercise. European Journal of Applied Physiology and Occupational Physiology, 36(1), 7–17. 1001318 10.1007/BF00421629

[phy270180-bib-0027] Leimbach, W. N., Jr. , Wallin, B. G. , Victor, R. G. , Aylward, P. E. , Sundlof, G. , & Mark, A. L. (1986). Direct evidence from intraneural recordings for increased central sympathetic outflow in patients with heart failure. Circulation, 73(5), 913–919. 10.1161/01.cir.73.5.913 3698236

[phy270180-bib-0028] Lew, L. A. , Liu, K. R. , & Pyke, K. E. (2021). Reliability of the hyperaemic response to passive leg movement in young, healthy women. Experimental Physiology, 106(9), 2013–2023. 10.1113/ep089629 34216162

[phy270180-bib-0029] Limberg, J. K. , Casey, D. P. , Trinity, J. D. , Nicholson, W. T. , Wray, D. W. , Tschakovsky, M. E. , Green, D. J. , Hellsten, Y. , Fadel, P. J. , Joyner, M. J. , & Padilla, J. (2020). Assessment of resistance vessel function in human skeletal muscle: Guidelines for experimental design, Doppler ultrasound, and pharmacology. American Journal of Physiology. Heart and Circulatory Physiology, 318(2), H301–H325. 10.1152/ajpheart.00649.2019 31886718 PMC7052621

[phy270180-bib-0030] Lind, L. , Johansson, K. , & Hall, J. (2002). The effects of mental stress and the cold pressure test on flow‐mediated vasodilation. Blood Pressure, 11(1), 22–27. 11926347 10.1080/080370502753543927

[phy270180-bib-0031] McDaniel, J. , Fjeldstad, A. S. , Ives, S. , Hayman, M. , Kithas, P. , & Richardson, R. S. (2010). Central and peripheral contributors to skeletal muscle hyperemia: Response to passive limb movement. Journal of Applied Physiology, 108(1), 76–84. 10.1152/japplphysiol.00895.2009 19910331 PMC2885076

[phy270180-bib-0032] McDaniel, J. , Hayman, M. A. , Ives, S. J. , Fjeldstad, A. S. , Trinity, J. D. , Wray, D. W. , & Richardson, R. S. (2010). Attenuated exercise induced hyperemia with age: Mechanistic insight from passive limb movement. The Journal of Physiology, 588, 4507–4517. 10.1113/jphysiol.2010.198770 20876201 PMC3008854

[phy270180-bib-0033] Messere, A. , Ceravolo, G. , Franco, W. , Maffiodo, D. , Ferraresi, C. , & Roatta, S. (2017). Increased tissue oxygenation explains the attenuation of hyperemia upon repetitive pneumatic compression of the lower leg. Journal of Applied Physiology, 123(6), 1451–1460. 10.1152/japplphysiol.00511.2017 28819006

[phy270180-bib-0034] Mitchell, J. H. , Reeves, D. R., Jr. , Rogers, H. B. , & Secher, N. H. (1989). Epidural anaesthesia and cardiovascular responses to static exercise in man. The Journal of Physiology, 417(1), 13–24. 2621589 10.1113/jphysiol.1989.sp017787 PMC1189252

[phy270180-bib-0035] Mortensen, S. P. , Askew, C. D. , Walker, M. , Nyberg, M. , & Hellsten, Y. (2012). The hyperaemic response to passive leg movement is dependent on nitric oxide: A new tool to evaluate endothelial nitric oxide function. The Journal of Physiology, 590(17), 4391–4400. 10.1113/jphysiol.2012.235952 22733658 PMC3473293

[phy270180-bib-0036] Neunteufl, T. , Heher, S. , Katzenschlager, R. , Wolfl, G. , Kostner, K. , Maurer, G. , & Weidinger, F. (2000). Late prognostic value of flow‐mediated dilation in the brachial artery of patients with chest pain. The American Journal of Cardiology, 86(2), 207–210. 10913483 10.1016/s0002-9149(00)00857-2

[phy270180-bib-0037] Notarius, C. F. , Ando, S. , Rongen, G. A. , & Floras, J. S. (1999). Resting muscle sympathetic nerve activity and peak oxygen uptake in heart failure and normal subjects. European Heart Journal, 20(12), 880–887. 10.1053/euhj.1998.1447 10329093

[phy270180-bib-0038] Pyke, K. , Green, D. J. , Weisbrod, C. , Best, M. , Dembo, L. , O'Driscoll, G. , & Tschakovsky, M. (2010). Nitric oxide is not obligatory for radial artery flow‐mediated dilation following release of 5 or 10 min distal occlusion. American Journal of Physiology. Heart and Circulatory Physiology, 298(1), H119–H126. 10.1152/ajpheart.00571.2009 19880668

[phy270180-bib-0039] Rector, T. S. , Bank, A. J. , De Bruyn, V. H. , Garr, M. D. , Kraemer, M. D. , & Kubo, S. H. (1993). Effects of norepinephrine on endothelium‐dependent vasodilation of forearm resistance vessels. Clinical Pharmacology and Therapeutics, 53(3), 374–379. 8453857 10.1038/clpt.1993.35

[phy270180-bib-0040] Remensnyder, J. P. , Mitchell, J. H. , & Sarnoff, S. J. (1962). Functional sympatholysis during muscular activity. Observations on influence of carotid sinus on oxygen uptake. Circulation Research, 11, 370–380. 10.1161/01.res.11.3.370 13981593

[phy270180-bib-0041] Richardson, R. S. , Poole, D. C. , Knight, D. R. , Kurdak, S. S. , Hogan, M. C. , Grassi, B. , Johnson, E. C. , Kendrick, K. F. , Erickson, B. K. , & Wagner, P. D. (1993). High muscle blood flow in man: Is maximal O2 extraction compromised? Journal of Applied Physiology, 75(4), 1911–1916. 8282650 10.1152/jappl.1993.75.4.1911

[phy270180-bib-0042] Rubanyi, G. M. , Lorenz, R. R. , & Vanhoutte, P. M. (1985). Bioassay of endothelium‐derived relaxing factor(s): Inactivation by catecholamines. American Journal of Physiology. Heart and Circulatory Physiology, 249(1), H95–H101. 10.1152/ajpheart.1985.249.1.H95 3874557

[phy270180-bib-0043] Saltin, B. , & Mortensen, S. P. (2012). Inefficient functional sympatholysis is an overlooked cause of malperfusion in contracting skeletal muscle. The Journal of Physiology, 590(24), 6269–6275. 10.1113/jphysiol.2012.241026 22988143 PMC3533189

[phy270180-bib-0044] Saltin, B. , Rådegran, G. , Koskolou, M. D. , & Roach, R. C. (1998). Skeletal muscle blood flow in humans and its regulation during exercise. Acta Physiologica Scandinavica, 162(3), 421–436. 10.1046/j.1365-201X.1998.0293e.x 9578388

[phy270180-bib-0045] Shields, K. L. , Broxterman, R. M. , Jarrett, C. L. , Bisconti, A. V. , Park, S. H. , & Richardson, R. S. (2021). The passive leg movement technique for assessing vascular function: The impact of baseline blood flow. Experimental Physiology, 106(10), 2133–2147. 10.1113/EP089818 34411365

[phy270180-bib-0046] Sinoway, L. , & Prophet, S. (1990). Skeletal muscle metaboreceptor stimulation opposes peak metabolic vasodilation in humans. Circulation Research, 66(6), 1576–1584. 10.1161/01.res.66.6.1576 2344665

[phy270180-bib-0047] Strange, S. (1999). Cardiovascular control during concomitant dynamic leg exercise and static arm exercise in humans. The Journal of Physiology, 514(1), 283–291. 10.1111/j.1469-7793.1999.283af.x 9831733 PMC2269052

[phy270180-bib-0048] Sundlof, G. , & Wallin, B. G. (1978). Human muscle nerve sympathetic activity at rest. Relationship to blood pressure and age. The Journal of Physiology, 274(1), 621–637. 625012 10.1113/jphysiol.1978.sp012170 PMC1282513

[phy270180-bib-0049] Thijssen, D. H. J. , de Groot, P. , Kooijman, M. , Smits, P. , & Hopman, M. T. E. (2006). Sympathetic nervous system contributes to the age‐related impairment of flow‐mediated dilation of the superficial femoral artery. American Journal of Physiology. Heart and Circulatory Physiology, 291(6), H3122–H3129. 10.1152/ajpheart.00240.2006 16844924

[phy270180-bib-0050] Trinity, J. D. , Amann, M. , McDaniel, J. , Fjeldstad, A. S. , Barrett‐O'Keefe, Z. , Runnels, S. , Morgan, D. E. , Wray, D. W. , & Richardson, R. S. (2010). Limb movement‐induced hyperemia has a central hemodynamic component: Evidence from a neural blockade study. American Journal of Physiology. Heart and Circulatory Physiology, 299(5), H1693–H1700. 10.1152/ajpheart.00482.2010 20802133 PMC3774478

[phy270180-bib-0051] Trinity, J. D. , Groot, H. J. , Layec, G. , Rossman, M. J. , Ives, S. J. , Morgan, D. E. , Gmelch, B. S. , Bledsoe, A. , & Richardson, R. S. (2015). Passive leg movement and nitric oxide‐mediated vascular function: The impact of age. American Journal of Physiology. Heart and Circulatory Physiology, 308(6), H672–H679. 10.1152/ajpheart.00806.2014 25576629 PMC4360052

[phy270180-bib-0052] Trinity, J. D. , Groot, H. J. , Layec, G. , Rossman, M. J. , Ives, S. J. , Runnels, S. , Gmelch, B. , Bledsoe, A. , & Richardson, R. S. (2012). Nitric oxide and passive limb movement: A new approach to assess vascular function. The Journal of Physiology, 590(6), 1413–1425. 10.1113/jphysiol.2011.224741 22310310 PMC3382331

[phy270180-bib-0053] Trinity, J. D. , Kwon, O. S. , Broxterman, R. M. , Gifford, J. R. , Kithas, A. C. , Hydren, J. R. , Jarrett, C. L. , Shields, K. L. , Bisconti, A. V. , Park, S. H. , Craig, J. C. , Nelson, A. D. , Morgan, D. E. , Jessop, J. E. , Bledsoe, A. D. , & Richardson, R. S. (2021). The role of the endothelium in the hyperemic response to passive leg movement: Looking beyond nitric oxide. American Journal of Physiology. Heart and Circulatory Physiology, 320(2), H668–H678. 10.1152/ajpheart.00784.2020 33306447 PMC8082797

[phy270180-bib-0054] Trinity, J. D. , McDaniel, J. , Venturelli, M. , Fjeldstad, A. S. , Ives, S. J. , Witman, M. A. H. , Barrett‐O'Keefe, Z. , Amann, M. , Wray, D. W. , & Richardson, R. S. (2011). Impact of body position on central and peripheral hemodynamic contributions to movement‐induced hyperemia: Implications for rehabilitative medicine. American Journal of Physiology. Heart and Circulatory Physiology, 300(5), H1885–H1891. 10.1152/ajpheart.00038.2011 21357514 PMC3283041

[phy270180-bib-0055] Turturici, M. , & Roatta, S. (2013). Inactivation of the mechani‐sensitive dilation upon repetitive mechanical stimulation of the musculo‐vascular network in the rabbit. Journal of Physiology and Pharmacology, 64(3), 299–308. 23959726

[phy270180-bib-0056] Venturelli, M. , Rossman, M. J. , Ives, S. J. , Weavil, J. C. , Amann, M. , Wray, D. W. , & Richardson, R. S. (2022). Passive leg movement‐induced vasodilation and exercise‐induced sympathetic vasoconstriction. Autonomic Neuroscience, 239, 102969. 10.1016/j.autneu.2022.102969 35259576 PMC9044344

[phy270180-bib-0057] Victor, R. G. , Pryor, S. L. , Secher, N. H. , & Mitchell, J. H. (1989). Effects of partial neuromuscular blockade on sympathetic nerve responses to static exercise in humans. Circulation Research, 65(2), 468–476. 10.1161/01.res.65.2.468 2752552

[phy270180-bib-0058] Wallin, B. G. , & Charkoudian, N. (2007). Sympathetic neural control of integrated cardiovascular function: Insights from measurement of human sympathetic nerve activity. Muscle & Nerve, 36(5), 595–614. 17623856 10.1002/mus.20831

[phy270180-bib-0059] Wan, H. Y. , Bunsawat, K. , & Amann, M. (2023). Autonomic cardiovascular control during exercise. American Journal of Physiology. Heart and Circulatory Physiology, 325(4), H675–H686. 10.1152/ajpheart.00303.2023 37505474 PMC10659323

[phy270180-bib-0060] Weggen, J. B. , Hogwood, A. C. , Decker, K. P. , Darling, A. M. , Chiu, A. , Richardson, J. , & Garten, R. S. (2023). Vascular responses to passive and active movement in premenopausal females: Comparisons across sex and menstrual cycle phase. Medicine and Science in Sports and Exercise, 55(5), 900–910. 10.1249/mss.0000000000003107 36728956

[phy270180-bib-0061] Witman, M. A. , Ives, S. J. , Trinity, J. D. , Groot, H. J. , Stehlik, J. , & Richardson, R. S. (2015). Heart failure and movement‐induced hemodynamics: Partitioning the impact of central and peripheral dysfunction. International Journal of Cardiology, 178, 232–238. 10.1016/j.ijcard.2014.10.044 25464261 PMC4314508

[phy270180-bib-0062] Wray, D. W. , Witman, M. A. H. , Ives, S. J. , McDaniel, J. , Trinity, J. D. , Conklin, J. D. , Supiano, M. A. , & Richardson, R. S. (2013). Does brachial artery flow‐mediated vasodilation provide a bioassay for NO? Hypertension, 62(2), 345–351. 10.1161/hypertensionaha.113.01578 23774225 PMC3775568

[phy270180-bib-0063] Yamada, Y. , Miyajima, E. , Tochikubo, O. , Matsukawa, T. , & Ishii, M. (1989). Age‐related changes in muscle sympathetic nerve activity in essential hypertension. Hypertension, 13(6_Pt_2), 870–877. 10.1161/01.hyp.13.6.870 2737724

